# Intraoperative oxygen concentration and neurocognition after cardiac surgery: study protocol for a randomized controlled trial

**DOI:** 10.1186/s13063-017-2337-1

**Published:** 2017-12-19

**Authors:** Shahzad Shaefi, Edward R. Marcantonio, Ariel Mueller, Valerie Banner-Goodspeed, Simon C. Robson, Kyle Spear, Leo E. Otterbein, Brian P. O’Gara, Daniel S. Talmor, Balachundhar Subramaniam

**Affiliations:** 1 Department of Anesthesia, Critical Care and Pain Medicine, Beth Israel Deaconess Medical Center, Harvard Medical School, 330 Brookline Ave, Boston, MA 02215 USA; 2 Division of General Medicine and Primary Care, Beth Israel Deaconess Medical Center, Harvard Medical School, 330 Brookline Ave, Boston, MA 02215 USA; 3 Division of Gastroenterology, Beth Israel Deaconess Medical Center, Harvard Medical School, 3 Blackfan Circle, Boston, MA 02215 USA; 4 Division of Cardiothoracic Surgery, Beth Israel Deaconess Medical Center, Harvard Medical School, 330 Brookline Ave, Boston, MA 02215 USA; 5 Division of Transplant Surgery, Center for Life Sciences, Beth Israel Deaconess Medical Center, Harvard Medical School, Boston, MA 02215 USA

**Keywords:** Hyperoxia, Neurocognition, Delirium, Normoxia, Cardiac surgery, Hyperoxemia, Coronary artery bypass grafting, Montreal Cognitive Assessment, Confusion Assessment Method, Oxygen therapy

## Abstract

**Background:**

Postoperative cognitive dysfunction (POCD) is a common complication of cardiac surgery. Studies have identified potentially injurious roles for cardiopulmonary bypass (CPB) and subsequent reperfusion injury. Cognitive dysfunction has also been linked to the deleterious effects of hyperoxia following ischemia-reperfusion injuries in several disease states, but there has been surprisingly little study into the role of hyperoxia in reperfusion injury after CPB. The potential for tightly regulated intraoperative normoxia to ameliorate the neurocognitive decline following cardiac surgery has not been investigated in a prospective manner. We hypothesize that the use of a protocolized management strategy aimed towards maintenance of an intraoperative normoxic level of oxygen, as opposed to hyperoxia, will reduce the incidence of POCD in older patients undergoing cardiac surgery.

**Methods/Design:**

One hundred patients aged 65 years and older undergoing non-emergency coronary artery bypass grafting surgery on cardiopulmonary bypass will be enrolled in this prospective, randomized, controlled trial. Subjects will be randomized to receive a fraction of inspired oxygen of either 35% or 100% while under general anesthesia throughout the intraoperative period. The primary outcome measure will be the incidence of POCD in the acute postoperative phase and up to 6 months. The assessment of neurocognition will be undertaken by trained personnel, blinded to study group, with the telephone Montreal Cognitive Assessment (t-MoCA) tool. Secondary outcome measures will include assessment of delirium using the Confusion Assessment Method (CAM and CAM-ICU), as well as time to extubation, days of mechanical ventilation, length of ICU and hospital stay and mortality at 6 months. With the aim of later identifying mechanistic aspects of the effect of oxygen tension, blood, urine, and atrial tissue specimens will be taken at various time points during the perioperative period and later analyzed.

**Discussion:**

This trial will be one of the first randomized controlled studies to prospectively assess the relationship between intraoperative oxygen levels and postoperative neurocognition in cardiac surgery. It addresses a promising biological avenue of intervention in this vulnerable aging population.

**Trial registration:**

ClinicalTrials.gov Identifier: NCT02591589, registered February 13, 2015.

**Electronic supplementary material:**

The online version of this article (doi:10.1186/s13063-017-2337-1) contains supplementary material, which is available to authorized users.

## Background

There are approximately 300,000 cardiac surgical procedures annually utilizing cardiopulmonary bypass (CPB) in the United States alone [1]. Postoperative cognitive dysfunction (POCD) remains a common adverse outcome in patients undergoing cardiac surgery, and has been reported to occur in up to 50% of patients after coronary artery bypass grafting (CABG) at the time of hospital discharge. Defined by deficits in working memory and executive function, this condition persists in as many as 30% of patients at 6 months [2]. A related cognitive disorder, postoperative delirium, also occurs frequently in this population. While often minimized due to its apparent temporary nature, recent data have demonstrated that postoperative delirium has lasting effects, with decreased cognitive performance up to 1 year after surgery and an accelerated rate of age-related cognitive decline compared to patients without delirium [3]. Despite the seemingly minor and temporary effects these cognitive deficits may seem to pose to the physical recovery of the cardiac surgical patient, data suggest that patients who suffer from these conditions display a higher rate of overall mortality [4]. Hence, postoperative delirium and POCD following cardiac surgery is a considerable problem, perhaps the most frequently encountered adverse complication following cardiac surgery [5]. The incidence of neurological sequelae after cardiac surgery is only expected to increase as surgery continues to be performed on an ever-aging population [6].

The delivery of oxygen has long been a cornerstone of anesthesia practice, traditionally with titration of oxygen therapy to ensure avoidance of potentially injurious periods of hypoxemia. However, the same attention has not been afforded for levels of relative hyperoxemia, presuming that excess oxygen is relatively harmless. Emerging clinical data within a variety of arenas suggest that this assumption may not be true. There appears to be potentially deleterious effects from hyperoxemia, whether it be demonstration of extension of infarct size post myocardial infarction [7], worse neurological outcomes and higher mortality in patients receiving therapeutic hypothermia following return of spontaneous circulation (ROSC) after cardiac arrest [8], or higher mortality in the intensive care unit [9]. Given these data, recent investigation has focused on assessment of potentially detrimental consequences of hyperoxemia in the domain of CPB during cardiac surgery [10, 11]. However, the potential for tightly regulated intraoperative normoxia to ameliorate POCD following cardiac surgery has not been investigated in a prospective manner [12, 13].

The objective of this trial is to determine whether patients undergoing CABG on CPB maintained under normoxic conditions throughout the intraoperative period will have a lower incidence of POCD and delirium than those exposed to hyperoxia. Given the disease burden, investigation into such a mechanistically intuitive and potentially simple intervention could impact hundreds of thousands of cardiac surgery patients a year.

## Methods/design

### Study design

**Fig. 1:**
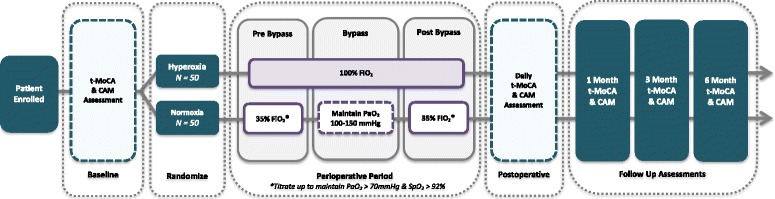
Study schema

To assess the relationship between intraoperative arterial partial pressure of oxygen (PaO_2_) and the incidence of POCD and delirium we have designed a single-center, randomized, double-blind superiority trial of 100 participants with an allocation of 1:1. Subjects undergoing CABG surgery with CPB will be prospectively randomized to one of two different levels of fraction of inspired oxygen (FiO_2_) intraoperatively. The primary outcome of postoperative cognitive decline will be assessed via cognitive testing at baseline, daily from postoperative day 1 until hospital discharge, and then by phone at 1, 3, and 6 months postoperatively (Fig. 1 for trial schematic). Table 1 details the structured study summary according to the World Health Organization Trial Registration Data Set. The trial protocol was written in accordance with the Standard Protocol Items: Recommendations for Interventional Trials (SPIRIT) checklist [14] (Additional file 1).

**Table 1:** WHO Trial Registration Data Set - Structured Summary

Data category	Information
Primary registry, trial identifying number	Clinicaltrials.gov Identifier - NCT02591589
Date of registration in primary registry	February 13, 2015
Secondary identifying numbers	
Sources of monetary support	Foundation for Anesthesia Education and Research Mentored Training Research Grant
Contact for public queries	SS, Department of Anesthesia, Beth Israel Deaconess Medical Center, Boston MA, USA
Contact for scientific queries	SS, Department of Anesthesia, Beth Israel Deaconess Medical Center, Boston MA, USA
Public title	Intraoperative Oxygen Concentration and Neurocognition After Cardiac Surgery
Scientific title	The relationship between administered oxygen levels and arterial partial oxygen pressure to neurocognition in cardiac surgical patients
Country of recruitment	USA
Health problem under investigation	Neurocognition after cardiac surgery
Key inclusion and exclusion criteria	Age ≥ 65 years, elective or urgent coronary artery bypass surgery (CABG) on cardiopulmonary bypass (CPB)
Study type	Interventional Allocation: randomized Interventional model: Masking: blinding of participant and outcomes assessor
Date of first enrollment	September 2015
Target sample size	100
Recruitment status	Recruiting as of January 2017
Primary outcome	Change in Telephonic Montreal Cognitive Assessment from baseline up to 6 months
Key secondary outcomes	Delirium incidence, time to extubation, Acute kidney injury incidence

**Fig. 2:**
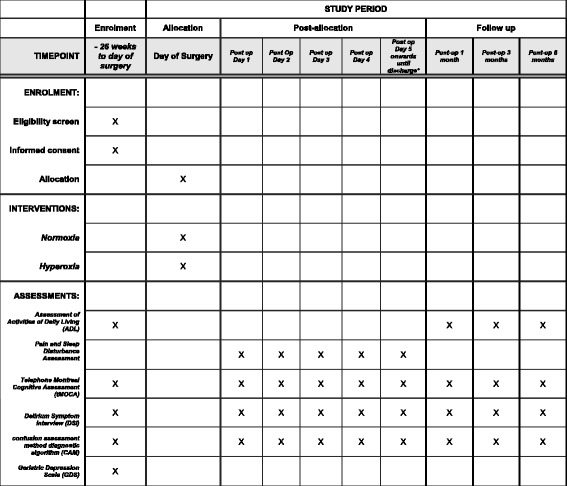
Standard Protocols Items: Recommendations for Interventional Trials (SPIRIT) figure

The SPIRIT figure (Fig. 2) demonstrates the schedule of enrollment, interventions, and instruments of assessment used in the trial.

### Study setting

Beth Israel Deaconess Medical Center (BIDMC) is a 700-bed tertiary care academic facility and Harvard Medical School teaching hospital. More than 900 open-heart procedures are performed on an annual basis, of which approximately 400 of these are CABG surgeries with CPB.

### Study registration

The study will be conducted in accordance with the ethical principles taken from the Declaration of Helsinki and adhere to the principles of Good Clinical Practice. The Committee on Clinical Investigations Institutional Review Board at Beth Israel Deaconess Medical Center approved the study protocol and provides ongoing oversight (IRB Protocol Number 2014-P-000398). Upon completion the trial will be reported in adherence to the Consolidated Standards of Reporting Trials (CONSORT) guidelines [15] in addition to the Standard Protocols Items: Recommendations for Interventional Trials (SPIRIT) checklist [14]. This study was registered at the U.S. National Institutes of Health with ClinicalTrials.gov on 13 February 2015 and carries the trial identification number NCT02591589. The trial is ongoing and currently recruiting. All amendments to the protocol are reported to the Institutional Review Board for approval.

### Inclusion and exclusion criteria

The study will enroll adult patients aged 65 or older undergoing elective or urgent on-bypass CABG cardiac surgery with subsequent admission to a cardiovascular intensive care unit (ICU). Patients undergoing emergent CABG surgery, combined CABG and other cardiac procedures, procedures requiring single lung ventilation, off-pump cardiac bypass surgery, or patients with signs of cardiogenic shock as dictated by preoperative inotropic, intra-aortic balloon counterpulsation or mechanical circulatory support devices will be excluded. Patients with evidence of severe cognitive impairment at baseline, as defined by a Telephone Montreal Cognitive Assessment (t-MoCA) score below 10, will also be excluded.

### Intervention and anesthesia regimen

Patients will be randomized on a 1:1 basis through a block randomization schedule using sealed envelopes to one of two experimental groups based on arterial oxygen tensions. In the first group, the FiO_2_ will be set at a minimum of 0.35 to maintain their PaO_2_ above 70 mmHg or oxygen saturations (SpO_2_) greater than or equal to 92%, and if necessary, titrated up to prevent potentially injurious hypoxemia (SpO_2_ < 92%). During CPB a blended air/oxygen mixture will be titrated to arterial blood gas analysis with aim of maintenance of PaO_2_ between 100 and 150 mmHg. This intervention group will be termed the normoxic arm.

The second arm of this trial will consist of subjects exposed to an FiO_2_ set at 1.0 throughout the intraoperative period, including during cardiopulmonary bypass. PaO_2_ will be measured periodically; however, it will not be used to titrate FiO_2_. This control group will be termed the hyperoxic arm.

Anesthetic regimen will not be standardized but is instead at the discretion of the treating provider. To standardize other key aspects of ventilator support in line with current best practice, mechanical ventilation will be performed according to a protocol based on current institutional standard of care. Tidal volumes will be set to 6–8 cc/kg and positive end-expiratory pressure (PEEP) levels of 0–5 cm H_2_O in both groups, with flexibility for provider preference. Study ventilator settings will be applied after induction of general anesthesia and successful endotracheal intubation and continued throughout the surgery, until surgical closure. Blood gas analysis and recording will be performed in both groups in the same manner as in the course of normal clinical care. Intraoperative FiO_2_, arterial oxygen saturation, mean arterial pressure, tidal volume, PEEP, and temperature will be extracted from the anesthesia record at 30-second intervals.

### Safety assessments and blinding

The Principal Investigator and other physician co-investigators will be in communication with the clinical team throughout the intra-operative period to ensure protocol adherence. Further criteria for withdrawal from the study preoperatively include active ongoing cardiac ischemia or acute decompensated arrhythmia, low oxygen saturation (SpO_2_ < 90%) on supplemental oxygen, or the use of continuous vasopressor or inotrope infusion medications. Patients will be withdrawn intraoperatively if they develop significant hemodynamic compromise because of cardiac surgery or sustained intra-operative oxygen desaturation (SpO_2_ < 90% for more than 3 minutes). At any time during the perioperative period patients may be withdrawn for significant physician or nursing concern.

Because real-time monitoring of protocol compliance requires knowledge of the patient’s assigned treatment, the study team utilizes a coordinated team approach. For every case, the study team members assessing the primary and secondary outcomes of the study will be blinded to the group assignment. This includes research staff administering neurocognitive assessments as well as physician staff monitoring patient progress and safety. The physician principal investigator is unblinded, and is responsible for conveying the treatment assignment to the clinical team and monitoring compliance with the assigned regimen intraoperatively. A trials specialist within the research group will monitor protocol compliance, occurrence and report any adverse events to the institutional review board (IRB).

## Study outcomes and their measurement

### Primary endpoint

To assess the occurrence of POCD we will use the t-MoCA. Scores on the t-MoCA will be measured preoperatively and postoperatively, using a daily in-hospital assessment until discharge or 30 days, whichever is sooner. In order to reduce fatigue completing assessments, if t-MoCA scores have plateaued for three consecutive days and CAM assessments are negative, assessments will occur every third day in-hospital through a skip pattern algorithm.

The t-MOCA is an adaptation of the widely used MoCA assessment [16], with items requiring writing and picture recognition removed. T-MoCA results in a 22-point scale and has been validated as a marker for cognitive function [16, 17]. Additionally, there will be telephone follow-up and t-MOCA assessment at 1, 3, and 6 months postoperatively, which does not require that participants be assessed in person. The telephonic version of the MoCA was chosen as the cognitive test to be performed both in-person and in telephonic follow-up so as to have equivalent scoring between assessments at all follow-up time points throughout the study. In previous studies, testing to 6 months has been shown to accurately reflect longitudinal follow-up of cognitive trajectories after CABG [18, 19] and thus this was the time endpoint for the follow-up in our study. Blinded, specially trained study staff will administer the assessments and assist in data collection.

### Secondary endpoints

The Confusion Assessment Method (CAM), which is a diagnostic algorithm for delirium, will be administered simultaneously with the t-MoCA postoperatively, as there are data to support a more pronounced reduction in cognitive ability in patients exhibiting postoperative delirium. This will assess for differential incidences of postoperative delirium that may occur between groups as a secondary endpoint. While patients are in the ICU and non-verbal, the t-MoCA will not be assessed and the validated CAM-ICU test for the incidence of delirium will be used in place of the CAM [19, 20]. Other secondary outcomes to be measured will include the time to extubation, days of mechanical ventilation, length of ICU and hospital stay, patient mortality at 30 days and 6 months. Other postoperative events as collated and reported in the Society of Thoracic Surgeons (STS) database will be reported and assessed including renal failure, stroke, myocardial infarction, reoperation, and sternal wound infection.

### Data collection

Clinical and demographic variables will be collected and reported including age, body mass index, ejection fraction and other aspects of advanced echocardiographic assessment, STS mortality score, and comorbidities (for example: history of myocardial infarction, congestive heart failure, diabetes, dyslipidemia, hypertension, stroke, peripheral vascular disease, and smoking from the predefined STS database Version 2.6). In addition, assessments will be made regarding functional Activities of Daily Living (ADL) and Geriatric Depression Scale (GDS).

All information collection will employ electronic case report forms, namely Research Electronic Data Capture [21]. REDCap is a secure, web-based application designed to support data capture for clinical trials that allows customized data collection fields to support individual trial needs. Members of the research team will be responsible for building and maintaining the electronic case report form (eCRF), as well as monitoring the data entry for completeness, timeliness and accuracy. Inter-rater reliability checks between multiple assessors will be performed throughout the study.

### Reporting of compliance and adverse events

A specialist within the research group will monitor protocol compliance, occurrence and reporting of adverse events to the IRB.

### Bio-specimen collection and processing

Biological specimens will be collected throughout the perioperative period. Blood, atrial tissue, and urine specimens will be collected intra-operatively, both at pre-bypass and post-bypass time points. Blood and urine specimens will also be collected at other time points in the perioperative period. These specimens will be used for analysis for biomarkers of oxidative stress and investigation of mechanistic pathways.

### Follow-up

Subjects will be contacted by telephone by trained study team members for 1, 3, and 6-month t-MoCA, ADL, and GDS assessments. For feasibility, the protocol allows for a time window of 7 days before and after the 1-month follow-up, and 14 days before or after the 3- and 6-month postoperative calls.

## Statistical analysis

### Sample size calculation

Using previous data [3], and assuming a cross walk between t-MoCA and Mini-Mental State Examination (MMSE) [22], a two-sided α = 0.05 and 80% power, we will need 74 subjects in total to detect a mean difference in t-MoCA scores of 2 (standard deviation of 3) between the hyperoxia and normoxia group at postoperative day 2. This cognitive decrement was previously reported as clinically significant and postoperative day 2 was reported as the in-hospital postoperative nadir time point [19]. A total of 100 randomized subjects who have undergone the entire intraoperative intervention will be enrolled to allow for potential attrition from loss to follow-up or withdrawal.

### Data analysis

Descriptive statistics of the data will be performed. Continuous data will be represented using mean (± standard deviation) or median (interquartile range) for variables not normally distributed and compared using parametric or non-parametric *t* tests as appropriate. Categorical data will be presented using proportions and compared using a chi-square (or Fisher’s exact) test. Two-sided *p* values less than 0.05 will be considered statistically significant. All analyses will be conducted using the intention-to-treat principle. No interim analyses are planned and there are no stopping rules for this trial. SAS 9.3 (SAS Institute, Cary, NC, USA) will be used for all analyses.

The primary outcome of the study is t-MoCA trajectories (values of t-MoCA) at postoperative day 2 between the two groups. An unadjusted *t* test (or non-parametric equivalent) will be used to assess superiority. Linear regression will be performed to identify independent predictors of t-MoCA as well as changes in t-MoCA over the 6-month study period. To account for the multiple measurements per subject, we will employ repeated measures regression techniques. Due to flexibility of timing of patient interviews, possibility of censored data and repeated observations, secondary analysis would require the use of a piecewise mixed-effects linear regression model measurement. Interaction will be assessed in the mixed-effects model to test if the t-MoCA slopes are different between groups.

Secondary analysis of adverse events including incidence of delirium, stroke, myocardial infarction, acute kidney injury (AKI), and hypoxia will also be assessed by employing logistic regression or log-binomial regression for outcomes with an incidence of 10% or greater. Odds ratios or measures of relative risk will include 95% confidence intervals. Secondary outcomes including ADL and GDS values will also be assessed using a trajectory analysis, similar to that employed for t-MoCA scores over time. The harvested biological specimens will provide mechanistic data regarding impact of administered oxygen levels on plasma, urine, and tissue biomarkers. To test this, a linear mixed model will be used to estimate the trajectories of values over the study period. The treatment-by-time interaction will quantify differences in trajectories between the groups. Initial modeling will include participant-specific slope and intercepts with likelihood ratio tests of these variance components considered for model parsimony.

### Protocol funding sources and their role

This study is supported by the Foundation for Anesthesia Education and Research (Mentored Training Research Grant). Funds have been allotted from this organization to support Principal Investigator time and effort. Additional funds have been awarded by the Beth Israel Deaconess Medical Center Chief Academic Officer Pilot Award for supporting study staff salary, statistical support, regulatory compliance consulting, and biospecimen processing. The scientific content of the study protocol and execution of the trial is in no way influenced by these funding sources. Protocol development, execution, and adherence, as well as scientific content development is supported under the Center for Anesthesia Research Excellence (CARE) within the Department of Anesthesia at Beth Israel Deaconess Medical Center.

## Discussion

### Significance and innovation

The biological plausibility of the potentially detrimental effects of hyperoxia on neurocognition and other end organ manifestations is clear although complex [23]. Our study is innovative in that it is one of the first to investigate the concept of strict oxygen regulation in patients undergoing CABG with cardiopulmonary bypass and its potential relationship to postoperative neurological outcome. If clinical benefit is shown, the titration of oxygen therapy towards tighter normoxia as opposed to the allowance of hyperoxia would represent an easily attainable practice change for more than 300,000 patients a year in the United States alone, and would also potentially serve as a low-cost, ubiquitous intervention with widespread potential use across many disciplines.

### Limitations

We are limited in our ability to comment on any differences we observed in relation to cognitive decline associated with normal aging. In addition, this study includes patients undergoing on-bypass CABG surgery alone. The homogenous nature of the surgical population allows for a close examination of the outcomes within each arm, although future work may include a wider range of valvular and combined cardiac surgical populations.

There are also several potential limitations associated with our measurement of the primary outcome. That is, despite promising results regarding the sensitivity and specificity of the t-MoCA assessment in a clinical scenario, it has not been rigorously validated in this particular patient population. We are limited in our ability to discuss the association between intraoperative oxygen administration and both visuospatial components and some of the executive cognitive domains, as t-MoCA does not measure them directly. The t-MoCA does, however, address memory, attention, language, abstraction, recall, and several other important components of neurocognitive function. Furthermore, it is possible that differences could occur between in-person assessments that occur over the phone [16]. In order to mitigate this potential bias, our assessors have been extensively trained on administration of the t-MoCA, with an emphasis on consistency. While we cannot completely eliminate this potential bias, be believe that this is a good step towards addressing differences.

Of note, a recent study with a similar intervention found some challenges in protocol adherence in the normoxia group [11]. This unanticipated finding will be examined specifically in our group’s analyses to ascertain the scope of this experience [24].

The greatest source of potential bias that we see in this study is the possibility of loss to follow-up. The effect size calculated is relatively large. In an effort to mitigate loss of power due to dropouts, we have incorporated the potential for dropout into our sample size calculation. Furthermore, we plan to use a validated telephonic method for follow-up, allowing ample time windows for completion of telephonic postoperative follow-up, to reduce the chance that this will bias the results. It is also possible that there will be fatigue in completing repeated postoperative assessments daily. As such we have incorporated a skip pattern, so that subjects with plateaued score and negative CAM assessments will complete the in-hospital assessments every 3 days instead of daily. We believe that incorporating these strategies will reduce potential loss to follow-up and assessment refusal.

There is emerging evidence that titration of oxygen towards stringent normoxic targets may be beneficial [8, 9, 25, 26] in various groups of patients subjected to life-threatening injury or illness. This is one of the first randomized controlled trials that prospectively evaluates the influence of oxygen titration on neurological outcome in cardiac surgery. Further studies will be required to examine the mechanisms underlying any biological effects of oxygen level on neurocognition.

### Trial status

The trial is ongoing and currently recruiting.

## Additional file

Additional file 1: Standard Protocol Items: Recommendations for Interventional Trials (SPIRIT) checklist. The document represents the SPIRIT Checklist. (DOC 121 kb)

## Abbreviations

ADL: Activities of Daily Living
AKI: Acute kidney injury
CABG: Coronary artery bypass graft
CAM: Confusion Assessment Method
CAM-ICU: Confusion Assessment Method – Intensive Care Unit
CARE: Center for Anesthesia Research Excellence
CONSORT: Consolidated Standards of Reporting Trials
CPB: Cardiopulmonary bypass
eCRF: Electronic case report form
FiO_2_: Fraction of inspired oxygen
GDS: Geriatric Depression Scale
ICU: Intensive care unit
IRB: Institutional Review Board
MMSE: Mini Mental Status Exam
PaO_2_: Arterial partial pressure of oxygen
PEEP: Positive-end expiratory pressure
POCD: Postoperative cognitive decline
REDCap: Research Electronic Data Capture
ROSC: Return of spontaneous circulation
SPIRIT: Standard Protocols Items: Recommendations for Interventional Trials
SpO_2_: Peripheral saturation of oxygen
STS: Society of Thoracic Surgeons
t-MoCA: Telephone Montreal Cognitive Assessment

## References

[1] Health, United States, 2015: With Special Feature on Racial and Ethnic Health Disparities. Hyattsville, MD. 2016.: National Center for Health Statistics; 2015. 27308685

[2] Newman MF, Kirchner JL, Phillips-Bute B, Gaver V, Grocott H, Jones RH, Mark DB, Reves JG, Blumenthal JA (2001). Longitudinal assessment of neurocognitive function after coronary-artery bypass surgery. N Engl J Med.

[3] Inouye SK, Marcantonio ER, Kosar CM, Tommet D, Schmitt EM, Travison TG, Saczynski JS, Ngo LH, Alsop DC, Jones RN (2016). The short-term and long-term relationship between delirium and cognitive trajectory in older surgical patients. Alzheimers Dement.

[4] Gottesman RF, Grega MA, Bailey MM, Pham LD, Zeger SL, Baumgartner WA, Selnes OA, McKhann GM (2010). Delirium after coronary artery bypass graft surgery and late mortality. Ann Neurol.

[5] Lombard FW, Mathew JP (2010). Neurocognitive dysfunction following cardiac surgery. Semin Cardiothorac Vasc Anesth.

[6] Bartels K, McDonagh DL, Newman MF, Mathew JP (2013). Neurocognitive outcomes after cardiac surgery. Curr Opin Anaesthesiol.

[7] Stub D, Smith K, Bernard S, Nehme Z, Stephenson M, Bray JE, Cameron P, Barger B, Ellims AH, Taylor AJ (2015). Air versus oxygen in ST-segment-elevation myocardial infarction. Circulation.

[8] Kilgannon JH, Jones AE, Shapiro NI, Angelos MG, Milcarek B, Hunter K, Parrillo JE, Trzeciak S, Emergency Medicine Shock Research Network Investigators (2010). Association between arterial hyperoxia following resuscitation from cardiac arrest and in-hospital mortality. JAMA.

[9] Girardis M, Busani S, Damiani E, Donati A, Rinaldi L, Marudi A, Morelli A, Antonelli M, Singer M (2016). Effect of conservative vs conventional oxygen therapy on mortality among patients in an intensive care unit: the oxygen-ICU randomized clinical trial. JAMA.

[10] Smit B, Smulders YM, de Waard MC, Boer C, Vonk AB, Veerhoek D, Kamminga S, de Grooth HJ, Garcia-Vallejo JJ, Musters RJ (2016). Moderate hyperoxic versus near-physiological oxygen targets during and after coronary artery bypass surgery: a randomised controlled trial. Crit Care.

[11] McGuinness SP, Parke RL, Drummond K, Willcox T, Bailey M, Kruger C, Baker M, Cowdrey KA, Gilder E, McCarthy L, Painter T (2016). A multicenter, randomized, controlled phase IIb trial of avoidance of hyperoxemia during cardiopulmonary bypass. Anesthesiology.

[12] Fontes MT, McDonagh DL, Phillips-Bute B, Welsby IJ, Podgoreanu MV, Fontes ML, Stafford-Smith M, Newman MF, Mathew JP, Neurologic Outcome Research Group of the Duke Heart Center (2014). Arterial hyperoxia during cardiopulmonary bypass and postoperative cognitive dysfunction. J Cardiothorac Vasc Anesth.

[13] Joachimsson PO, Sjoberg F, Forsman M, Johansson M, Ahn HC, Rutberg H (1996). Adverse effects of hyperoxemia during cardiopulmonary bypass. J Thorac Cardiovasc Surg.

[14] Chan AW, Tetzlaff JM, Altman DG, Laupacis A, Gotzsche PC, Krleza-Jeric K, Hrobjartsson A, Mann H, Dickersin K, Berlin JA (2013). SPIRIT 2013 statement: defining standard protocol items for clinical trials. Ann Intern Med.

[15] Schulz KF, Altman DG, Moher D (2010). CONSORT 2010 statement: updated guidelines for reporting parallel group randomized trials. Ann Intern Med.

[16] Pendlebury ST, Welch SJ, Cuthbertson FC, Mariz J, Mehta Z, Rothwell PM (2013). Telephone assessment of cognition after transient ischemic attack and stroke: modified telephone interview of cognitive status and telephone Montreal Cognitive Assessment versus face-to-face Montreal Cognitive Assessment and neuropsychological battery. Stroke.

[17] Aykut K, Albayrak G, Guzeloglu M, Baysak A, Hazan E (2013). Preoperative mild cognitive dysfunction predicts pulmonary complications after coronary artery bypass graft surgery. J Cardiothorac Vasc Anesth.

[18] Cormack F, Shipolini A, Awad WI, Richardson C, McCormack DJ, Colleoni L, Underwood M, Baldeweg T, Hogan AM (2012). A meta-analysis of cognitive outcome following coronary artery bypass graft surgery. Neurosci Biobehav Rev.

[19] Saczynski JS, Marcantonio ER, Quach L, Fong TG, Gross A, Inouye SK, Jones RN (2012). Cognitive trajectories after postoperative delirium. N Engl J Med.

[20] Rudolph JL, Inouye SK, Jones RN, Yang FM, Fong TG, Levkoff SE, Marcantonio ER (2010). Delirium: an independent predictor of functional decline after cardiac surgery. J Am Geriatr Soc.

[21] Harris PA, Taylor R, Thielke R, Payne J, Gonzalez N, Conde JG (2009). Research electronic data capture (REDCap)--a metadata-driven methodology and workflow process for providing translational research informatics support. J Biomed Inform.

[22] Saczynski JS, Inouye SK, Guess J, Jones RN, Fong TG, Nemeth E, Hodara A, Ngo L, Marcantonio ER (2015). The Montreal Cognitive Assessment: creating a crosswalk with the Mini-Mental State Examination. J Am Geriatr Soc.

[23] Hafner S, Beloncle F, Koch A, Radermacher P, Asfar P (2015). Hyperoxia in intensive care, emergency, and peri-operative medicine: Dr. Jekyll or Mr. Hyde? A 2015 update. Ann Intensive Care.

[24] Shaefi S, Talmor DS, Subramaniam B (2016). Oxygen therapy: when is too much too much?. Anesthesiology.

[25] Helmerhorst HJ, Roos-Blom MJ, van Westerloo DJ, de Jonge E (2015). Association between arterial hyperoxia and outcome in subsets of critical illness: a systematic review, meta-analysis, and meta-regression of cohort studies. Crit Care Med.

[26] de Jonge E, Peelen L, Keijzers PJ, Joore H, de Lange D, van der Voort PH, Bosman RJ, de Waal RA, Wesselink R, de Keizer NF (2008). Association between administered oxygen, arterial partial oxygen pressure and mortality in mechanically ventilated intensive care unit patients. Crit Care.

